# Application of qPCR in conjunctival swab samples for the evaluation of canine leishmaniasis in borderline cases or disease relapse and correlation with clinical parameters

**DOI:** 10.1186/s13071-014-0460-3

**Published:** 2014-10-21

**Authors:** Marcello Ceccarelli, Luca Galluzzi, Davide Sisti, Barbara Bianchi, Mauro Magnani

**Affiliations:** Department of Biomolecular Sciences, University of Urbino “Carlo Bo”, via Saffi 2, 61029 Urbino, PU Italy; Veterinary Clinic “Santa Teresa”, via Piave 23, 61032 Fano, PU Italy

**Keywords:** *Leishmania infantum*, Conjunctival swabs, qPCR, Dog, Canine leishmaniasis, IFAT, Haemoglobin, Globulins

## Abstract

**Background:**

In leishmaniasis caused by *Leishmania infantum*, the dog acts as the main reservoir for the disease. Non-invasive sampling for *Leishmania* detection is pivotal for rapid and affordable diagnosis. Recently, the use of conjunctival swab (CS) has been evaluated as a non-invasive sampling technique for quantitative real-time PCR (qPCR). However, few investigations have been made on the applicability of CS qPCR in particular cases such as dogs with borderline IFAT titres, suspected disease relapse with comorbidity and therapy monitoring. The aims of this study were i) to confirm the efficacy of CS, comparing these samples to buffy coat (BC) samples, as effective non-invasive samples for *Leishmania* quantitative detection by qPCR and ii) to verify the usefulness of qPCR compared to conventional laboratory and clinical parameters to assist in therapeutic decision making regarding dogs with complex clinical cases.

**Methods:**

Eighty dogs were divided into 4 groups based on their IFAT titres and clinical histories. Two qPCR assays were performed both on CS raw lysates and on purified DNA from BC samples. The assays were then compared. Z tests for difference of proportion, with Bonferroni correction, were carried out to evaluate the qPCR results. Logistic regression with backward stepwise elimination was performed to detect the subset of haematochemical variables significantly associated with PCR positivity.

**Results:**

The qPCR performed on CS samples showed better sensitivity (87%) and specificity (96%) than assays carried out using BC samples, regardless of the primers used. The haematochemical parameters haemoglobin and globulins were found to be significantly associated with qPCR positivity. Pearson correlations between *Leishmania k*DNA load in CS and body condition scores or IFAT titres were calculated in dogs with new leishmaniasis diagnoses. The *Leishmania k*DNA load in CS correlated moderately with IFAT titres (R = 0.59) but a very weak correlation (R = 0.37) with body condition score (BCS) was found.

**Conclusions:**

The applicability of CS for *Leishmania* detection in dogs was confirmed, revealing the usefulness of raw lysates for quantitative purposes. Moreover, the qPCR was found to be particularly useful in cases lacking a clear clinical diagnosis, where the haematochemical values cannot be predictive.

**Electronic supplementary material:**

The online version of this article (doi:10.1186/s13071-014-0460-3) contains supplementary material, which is available to authorized users.

## Background

Leishmaniasis is a parasitosis caused by at least 15 *Leishmania* species. The parasite, primarily transmitted by phlebotomine sand flies, cause 0.2–0.4 million cases of visceral leishmaniasis (VL) and 0.7–1.2 million cases of cutaneous leishmaniasis (CL) per year. Currently, 12 million people are affected by the disease in 98 countries [1].

Specifically, *Leishmania infantum* is the aetiological agent of zoonotic leishmaniasis, which is endemic in the Mediterranean Basin, Central America, South America and parts of Asia [2]. The dog is highly susceptible to *L. infantum* infection and is the main reservoir for this parasite. In light of this fact, the canine leishmaniasis (CanL) is acknowledged as a very important problem in veterinary medicine [3,4] and human epidemiology, also because many infected dogs are asymptomatic [5,6]. Although many diagnostic systems have been developed, the gold standard in the diagnosis of CanL is still open to debate [7,8]. The indirect fluorescence antibody test (IFAT) is considered as a reference method by the World Organization for Animal Health (OIE-Office International des Epizooties) among serological techniques [9]. Nevertheless, these techniques are not always reliable in the periods of prepatent infection, remission stages and in the latent forms of the disease like in “cryptic leishmaniasis” [10]. Moreover, particularly in Central and South America, IFAT can have cross-reactivity with other pathogens like *Trypanosoma cruzi*, *Trypanosoma caninum*, *Leishmania braziliensis*, and *Ehrlichia canis* [11]. In addition, in areas where the pathogen is endemic, uncertain IFAT titres ranging from 1:40 to 1:80 (below the positivity threshold: titre ≥ 1:160), are found in a significant number of animals. These titres, defined also as “borderline titres” [12], may be difficult to interpret by veterinary practitioners. In this context, molecular techniques can improve the diagnosis of leishmaniasis. In particular, qualitative and quantitative PCR (qPCR) may play an important role in parasite detection [13] and therapy monitoring [14,15], in both human and in veterinary medicine. In molecular methods, the constant region of *Leishmania* minicircles has been widely used as a specific target because of its very high number of target sequences [16-19]. Moreover, splenic aspirates and bone marrow are currently considered the clinical samples which yield the best sensitivity results [20,21]. Unfortunately, these samples are obtained with invasive procedures and usually require a general anaesthesia. The use of DNA purified from conjunctival swabs (CS) for PCR-based diagnosis of CanL was introduced in order to reduce the need for invasive procedures [22,23]. Moreover, Peña *et al.* [24] characterized the diffusion of the *Leishmania* parasite in different ocular tissues of infected dogs. More recently, Lombardo *et al.* [25] verified that CS have a sensitivity similar to that of lymph node samples, while de Almeida *et al.* [26] showed comparable PCR sensitivity in CS and bone marrow. Furthermore, the usefulness of CS PCR as diagnostic tool has also been confirmed in non-endemic areas [27]. Contextually, the sensitivity and utility of whole blood or buffy coat (BC) in veterinary medicine are still much disputed. In fact, PCR-based *Leishmania* detection in CanL using whole blood or buffy coat is not without difficulties [18]. It showed results variability [23,26-29], and is considered of little diagnostic value [30]. On the other hand, reliable therapy monitoring requires parasite quantification that may be difficult to achieve with the CS samples. In fact, CS analysis can be affected by variations in the amount of biological material sampled, which can vary among different samples from the same patient (for example pre-treatment and treatment phases). The usefulness of CS has been evaluated in the early diagnosis of leishmaniasis, but few works investigated their utility in the therapy monitoring [13]. Moreover, this approach has not been thoroughly investigated as a possible tool that could help guide treatment decisions in borderline cases, when there is suspicion of disease relapse or comorbidity. In the present investigation, we confirmed the usefulness of qPCR as a quantitative diagnostic tool using CS raw lysate samples as a source of DNA, particularly in cases with borderline IFAT titres, suspected disease relapse or the presence of comorbidity. Moreover, we identified the haematochemical parameters significantly associated with qPCR positive results.

## Methods

### Ethical statement

The study was approved by the Ethical Committee for Animal Experiments of the University of Urbino (CESA) on July 31st 2012. The study’s title was “Diagnosi biomolecolare della leishmaniosi attraverso l’uso di campioni clinici non invasivi e loro utilizzo per il monitoraggio terapeutico” (Prot. CESA 2/2012).

### Dogs

The study was carried out in the Marches region (Central Italy), where *L. infantum* is present as a parasite. A total of 80 dogs were enrolled (45 males, 35 females). The dogs either lived in kennels or with their owners. Two of the dogs were of unknown breed, 24 were mongrels and 54 were pure breeds. The median age of 51 dogs was 6 years (range: 2 months −15 years); the remaining 29 dogs were adults of unknown age. The dogs were examined by veterinary practitioners for the presence of signs attributable to *Leishmania* infection and scored for clinical signs from 0 to 14. A single point was attributed for each of the following 14 clinical signs: alopecia, dermatitis, skin ulceration, ocular lesions, dysorexia, weight loss, asthenia, vomiting and/or diarrhoea, epistaxis, hyperthermia, lymphadenopathy, lameness, onychogryposis and other (eventually specified). Dogs with a body condition score (BCS) of 0 were defined as asymptomatic; those with a score of 1–2, paucisymptomatic; those with a score of 3–6, oligosymptomatic; and animals with a score of 7–14, polisymptomatic.

In light of the IFAT results (see below) and considering their clinical history, the dogs were divided into 4 groups: A) IFAT negative dogs (n = 24), composed of healthy dogs (n = 14 with a clinical score of 0) and also symptomatic dogs suspected of having leishmaniasis by veterinary practitioners (n = 10 with clinical score 1–8); B) suspected for leishmaniasis (IFAT from 1:40 to 1:80; n = 17); C) dogs monitored after successful therapy (IFAT ≥ 1:160 at the time of first diagnosis; n = 16), composed of dogs that were diagnosed with leishmaniasis before the beginning of this study and brought to the veterinary clinic for a re-evaluation, in some cases because of the reappearance of symptoms (clinical score from 0 to 6); D) newly positive dogs (IFAT ≥ 1:160; n = 23). A further subgroup (group E) comprised dogs monitored during therapy (IFAT ≥ 1:160; n = 4). This group included dogs with qPCR results available both at the time of diagnosis and during therapy (dog 57, also included in group C; dogs 67, 68, 72, also included in group D).

### Canine samples

Canine peripheral blood (drawn for routine clinical tests) and conjunctival swabs (CS) were provided by the “Santa Teresa” Veterinary Clinic (Fano, Italy). Serum for biochemical assays and protein electrophoresis was obtained after the centrifugation of peripheral blood collected in a tube with serum-granules clotting activator at 1,900 × g for 5 min. The separated serum was stored at −20°C until it was analyzed. The whole blood (1 ml) for haematological assays was collected in tubes with EDTA and immediately analyzed. The buffy coat samples (100 to 320 μl) were obtained after centrifugation of residual peripheral blood at 210 × g for 10 min. Conjunctival swabs were collected from the right and left conjunctivas using sterile cotton swabs. The swabs were rubbed with a rotational movement back and forth three times in the lower conjunctival sac.

IFAT was performed on serum samples with an in-house assay validated and provided by the Institute of Experimental Preventive Veterinary Medicine (Istituto Zooprofilattico Sperimentale) of Sicily (IZS) [30]. In accordance with Gradoni *et al.* [12], IFAT titres below 1:40 were considered negative, those from 1:40 to 1:80 were defined as borderline and those ≥1:160 were considered positive.

### Clinical chemistry and haematological assays

Protein and albumin were quantified using an automated spectrophotometer (Pronto Evolution, BPC BioSed, Roma, Italy) and Giesse Diagnostics commercial kits (Giesse Diagnostics srl, Rome, Italy), based on the biuret and bromocresol green methods, respectively. Protein fractions were measured in 25 μl of serum by electrophoresis on cellulose acetate membranes (Auto Phor 400, Bio Group Medical System s.r.l., Talamello, Italy) and buffer (pH 8.8; Auto Phor 400, Bio Group Medical System s.r.l., Talamello, Italy) in a semi-automated electrophoresis analyzer (AdaLya 24, Seleo Engineering s.r.l., Orta di Atella, Italy) according to the manufacturer’s instructions. The haematological assays where performed using the coulter system Genius Vet haematology analyzer (Seac srl Radim Group, Calenzano, Italy).

### DNA extraction

The DNA was extracted from buffy coat and conjunctival samples as previously described [31]. Briefly, the DNA from buffy coat samples was extracted using the DNeasy Blood & Tissue kit (Qiagen), while the DNA from swabs was extracted by incubation for 2 h at 56°C in 200 μl lysis buffer (10 mM Tris–HCl pH 8.3, 50 mM KCl, 0.5% Nonidet P40, 0.5% Tween 20, 0.1 mg/ml proteinase K). After swab elimination, the samples were incubated for 10 min at 95°C and centrifuged at 14,000 × g for 10 min. Supernatants (raw lysates) were used directly as templates in PCR reactions.

### Quantitative PCR (qPCR) assays

Two qPCR assays were performed using two different primer pairs (MaryF-MaryR and MLF-MLR primers for qPCR1 and qPCR2, respectively) as previously reported [31]. Both primer pairs targeted the constant region of *L. infantum* kDNA minicircle. The standard curve was established using serial dilutions of Chelex-purified *L. (L.) infantum* MHOM/TN/80/IPT1 DNA, ranging from 100 to 0.001 parasites equivalent.

In CS samples, for quantitative purposes and to exclude false negative results due to low DNA extraction efficiency and/or the presence of PCR inhibitors, the canine beta-2-microglobulin (B2M) gene was used as a reference (qPCR3). In fact, quantification of the B2M gene was used to estimate the number of cells withdrawn in CS samples and consequently to normalize the *Leishmania* parasites to the number of canine cells. The primers B2Mcanis_F (5′-GTCCCACAGATCCCCCAAAG-3′) and B2Mcanis_R (5′- CTGGTGGATGGAACCCTGAC-3′) were used under the same conditions of qPCR1 and qPCR2. The B2M standard curve was constructed with serial dilutions of the B2M PCR product quantified using Qubit fluorometer (Life Technologies). In addition to the parasite quantification, a ΔCt value between *Leishmania* and B2M amplification curves (ΔCt Leish-B2M) was evaluated as an additional parameter to monitor parasite load changes in the same animal during the course of the illness or treatment. In fact, increases or decreases in ΔCt Leish-B2M correlate with decreases or increases in parasite loads, respectively.

### Statistical analysis

Descriptive statistics were performed with quantitative variables; PCR quantifications were log10 transformed in order to meet assumption of normal distribution. Analysis of variance (ANOVA) was used to test the associations between dog groups, clinical chemistry and haematological values. Z tests for proportions were performed in order to compare specificity and sensitivity values. The area under the receiver operating characteristic curve (AUC) allows an evaluation of the overall performance of a test or classification, i.e. its diagnostic accuracy; reported effect size intervals for AUC are: small effect sizes (0.528–0.556); medium effect sizes (0.584–0.638); large effect sizes (0.714–0.760); very large effect sizes (above 0.760) [32]. A good description of classification accuracy can be obtained by plotting an ROC (receiver operating characteristic) curve. The curve shows the probability of detecting true positives (sensitivity) and false positives (1-specificity) for an entire range of possible cut-points. Logistic regression with backward stepwise elimination were performed to detect the subset of first-line haematochemical variables significantly associated with PCR positivity. Every predictive variable (platelets, globulins, ratio albumin/globulins, white blood cells, red blood cells, haemoglobin, albumin) was dichotomized in 0 = value in physiological range and 1 = value out of physiological range. The Nagelkerke R square was reported as fitting index. The odds ratio and relative 95% confidence interval were reported for each predictive variable. All data were coded and analyzed using the Statistical Package for Social Science (SPSS) for Windows (Chicago, IL 60606, USA), version 13, or Excel 2003 (Microsoft_Office).

## Results

### IFAT, clinical chemistry and haematological parameters

Clinical parameters at the time of sampling for molecular assays for all the dog groups in our study are shown in Table 1. Clinical parameters values of all animals are supplied as supplementary materials (Additional file 1: Table S1). Serum electrophoresis was available for 47 dogs only (Additional file 2: Table S2). Such parameters did not provide significant information to decide whether to include dogs from groups B and C in the group of infected dogs requiring anti-*Leishmania* therapy (ANOVA test; p > 0.05). Therefore, the qPCR assays were used to detect and quantify the *Leishmania* parasites in CS and BC samples.

**Table 1:** **IFAT, body condition score (BCS), clinical chemistry and haematological mean values for all groups**

**Group (Dog ID)**		**IFAT**	**BCS**	**Prot (g/dl)**	**Alb (g/dl)**	**Glob (g/dl)**	**Ratio Alb/Glob**	**WBC (10** ^**3**^ **/μl)**	**RBC (10** ^**6**^ **/μl)**	**Hgb (g/dl)**	**Hct (%)**	**Plt (10** ^**3**^ **/μl)**
A(1–24)	mean ± s.e.	-	1.04 ± 0.37	6.49 ± 0.23	3.43 ± 0.14	3.05 ± 0.09	1.17 ± 0.14	15.34 ± 2.91	5.82 ± 0.27	15.28 ± 0.72	41.14 ± 1.81	292.83 ± 19.98
	min	<1/40	0	5.67	2.64	2.09	0.71	8.6	2.4	6.3	19.2	198
	max	<1/40	8	7.96	4.0	4.46	1.77	62.0	7.87	20.3	52.9	470
B(25–41)	mean ± s.e.	-	1.82 ± 0.52	6.86 ± 0.26	3.02 ± 0.06	3.85 ± 0.24	0.81 ± 0.05	11.95 ± 1.39	5.48 ± 0.22	14.22 ± 0.56	38.32 ± 1.30	347.55 ± 26.43
	min	1:40	0	5.86	2.8	2.96	0.55	6.2	4.30	11.1	31.1	185
	max	1:80	8	8.16	3.5	5.26	1.06	23.0	6.90	18.3	45.6	470
C(42–57)	mean ± s.e.	-	1.69 ± 0.49	6.63 ± 0.20	3.00 ± 0.13	3.63 ± 0.17	0.84 ± 0.06	9.88 ± 2.30	5.29 ± 0.62	14.19 ± 1.69	37.73 ± 4.30	258.08 ± 40.30
	min	1:80	0	5.83	1.9	2.53	0.45	4.2	0.92	2.7	7.4	106
	max	1:1280	6	8.35	3.9	5.15	1.30	28.7	7.14	19.4	50.7	523
D(58–80)	mean ± s.e.	-	3.61 ± 0.58	8.18 ± 0.33	2.48 ± 0.14	5.71 ± 0.39	0.54 ± 0.09	10.89 ± 1.06	5.02 ± 0.31	12.09 ± 0.73	34.32 ± 2.13	207.63 ± 22.58
	min	1:160	0	5.60	0.96	1.90	0.13	3.2	1.94	5.5	15.0	47
	max	1:10240	11	10.84	3.7	8.52	1.95	19.0	6.87	17.9	48.7	383

Prot: total proteins; Alb: albumins; Glob: globulins; WBC: white blood cells; RBC: red blood cells; Hgb: haemoglobin; HCT: haematocrit; Plt: platelets; s.e.: standard error.

### qPCR from conjunctival swabs (CS qPCR) and from buffy coat (BC qPCR)

**Figure 1:**
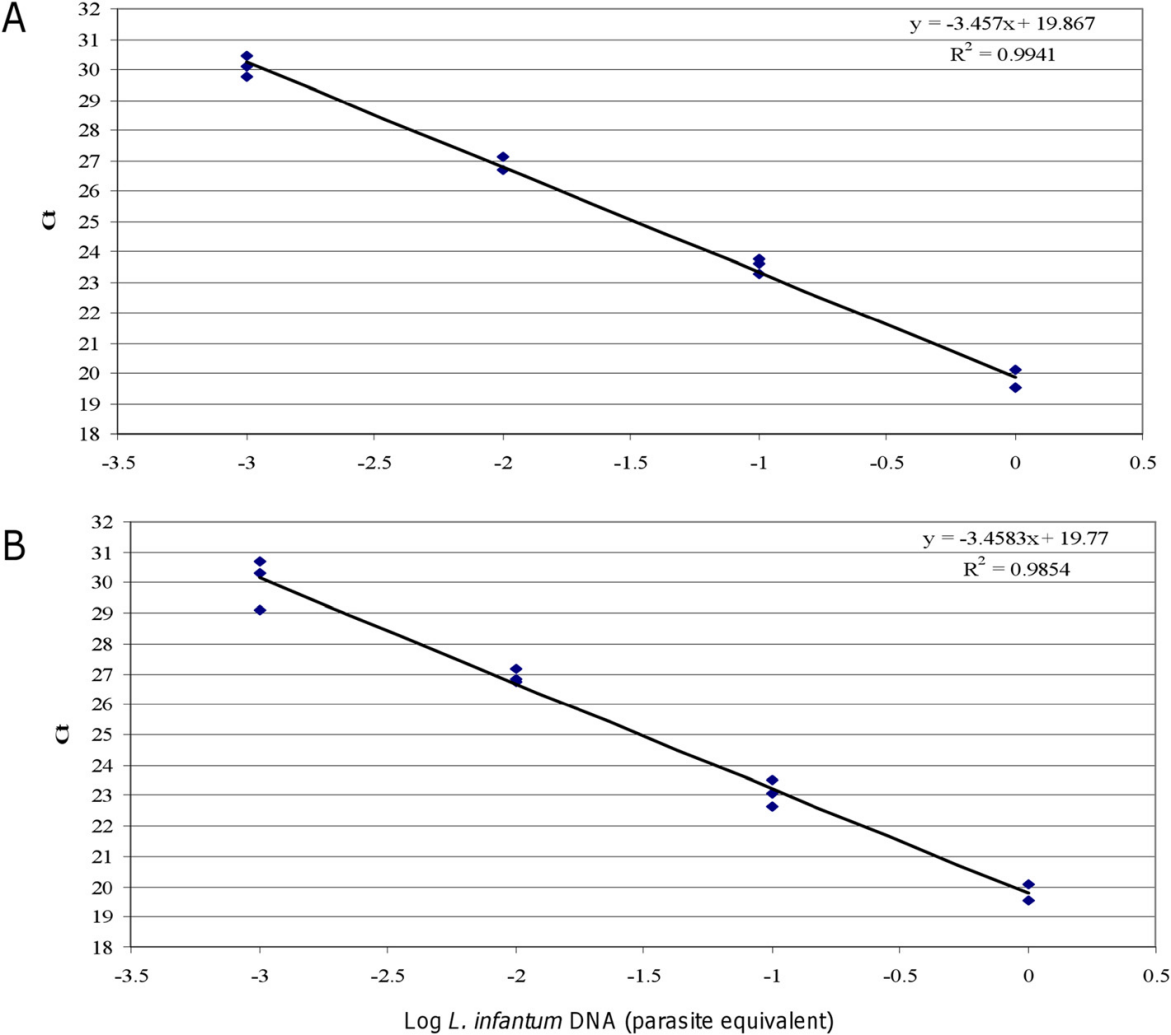
**qPCR1 sensitivity without (A) or with (B) raw CS lysate.** Serial dilutions of MHOM/TN/80/IPT1 *L. infantum* DNA (ranging from 1 to 0.001 parasite equivalent) were amplified in triplicate (1 μl in a total of 25 μl reaction mixture) **(panel A)**. To evaluate the eventual inhibitory effects, 1 μl of raw lysate was spiked in the PCR tubes containing the dilutions of MHOM/TN/80/IPT1 *L. infantum* DNA described above **(panel B)**.

The presence of *Leishmania* parasites was assessed in the dogs assigned to all groups by qPCR both from conjunctival swabs (CS qPCR) and buffy coat (BC qPCR). The qPCRs were initially performed with MaryF-MaryR primers (qPCR1); the positive results were subsequently confirmed using MLF-MLR primers (qPCR2). The amount of CS raw lysates to be used as a template in qPCR without any inhibitory effects was established as 1 μl per 25 μl reaction tube, maintaining the previously reported sensitivity (1×10^−3^ par/reaction tube) [31] (Figure 1). This raw lysate volume was used for all samples.

To allow normalization of the CS parasite load and to further exclude false negative results, all qPCR positive CS samples and part (34.5%) of the qPCR negative CS samples were tested for B2M amplification. In order to quantify the B2M copy number, a calibration curve was constructed using serial dilution of a purified PCR product (Additional file 3: Figure S1). On the whole, the B2M Ct average in CS samples was 23.26 ± 3.26 (n = 97 samples tested in duplicate). Assuming that B2M gene is present in two copies per diploid cell, the average Ct value corresponded to 49,950 cells per CS total raw lysate. This cell content was extremely variable from sample to sample, ranging from a minimum of 130 to a maximum of 2.34 × 10^6^ cells, with a median value of 75,590 cells, hence the need for a reference gene for normalization. The final CS qPCR results were expressed as the number of parasites normalized to 50,000 canine cells. The overall results of qPCR assays are reported in Table 2. Briefly, in group A 5 dogs were BC-positive and one of them was also CS-positive; in group B, one dog was BC-positive and 2 were CS-positive; in group C, 6 were BC-positive and 3 CS-positive; in group D, 20 and 14 dogs were found positive in CS and BC samples, respectively. The differences observed in the parasite quantifications between the two qPCR assays probably reflected the variability of minicircle subpopulations in the endemic *Leishmania* strains, compared to the reference strain MHOM/TN/80/IPT1 used to construct the standard curve [31].

**Table 2:** ***Leishmania*** **parasite quantification in buffy coat (BC) and conjunctival swab (CS) samples, by qPCR1 (normal text) and by qPCR2 (bold text)**

**Group**	**IFAT titre**	**Dog ID**	**BC qPCR (par/ml)**	**Left CS qPCR (par/5** **×10** ^**4**^ **cells)**	**Right CS qPCR (par/5×** **10** ^**4**^ **cells)**
A	< 1:40	1	0.01	0.00	0.00
			**n.a.**	**n.a.**	**n.a.**
		2, 5	0.00	n.a.	n.a.
			**n.a.**	**n.a.**	**n.a.**
		4	22.27	97.84	9.65
			**10.40**	**23.16**	**0.00**
		6	0.04	n.a.	n.a.
			**n.a.**	**n.a.**	**n.a.**
		7	0.03	0.00	0.00
			**n.a.**	**n.a.**	**n.a.**
		12	4.26	0.00	0.00
			**6.45**	**n.a.**	**n.a.**
		3, 8, 9, 10, 11, 13, 14, 15, 16, 17, 18, 19, 20, 21, 22, 23, 24	0.00	0.00	0.00
			**n.a.**	**n.a.**	**n.a.**
B	1:40 – 1:80	25, 28, 29, 30, 31, 32, 33, 34, 35, 36, 37, 38, 41	0.00	0.00	0.00
			**n.a.**	**n.a.**	**n.a.**
		26	0.07	0.00	0.00
			**n.a.**	**n.a.**	**n.a.**
		27	n.a.	0.05	0.00
			**n.a.**	**0.15**	**0.00**
		39	0.00	0.00	n.a.
			**n.a.**	**n.a.**	**n.a.**
		40	0.00	0.01	0.06
			**n.a.**	**0.06**	**0.89**
C	≥ 1:160	42	0.00	0.08	0.00
			**n.a.**	**n.a.**	**n.a.**
		43	0.7	n.a.	n.a.
			**n.a.**	**n.a.**	**n.a.**
		44	1.16	0.00	0.00
			**0.00**	**n.a.**	**n.a.**
		45	0.10	86.50	3.55
			**0.14**	**688.97**	**36.35**
		46	0.43	0.00	0.00
			**4.45**	**n.a.**	**n.a.**
		47	0.00	0.00	0.02
			**n.a.**	**n.a.**	**n.a.**
		48	0.21	0.00	0.00
			**0.00**	**n.a.**	**n.a.**
		49, 51, 52, 53, 54, 55, 56	0.00	0.00	0.00
			**n.a.**	**n.a.**	**n.a.**
		50	0.07	0.00	0.00
			**n.a.**	**0.00**	**0.00**
		57	0.00	0.00	1.83
			**n.a.**	**n.a**	**3.66**
D	≥ 1:160	58	0.52	0.00	0.00
			**3.40**	**0.00**	**0.00**
		59	1.49	n.a.	n.a.
			**5.00**	**n.a.**	**n.a.**
		60	2.83	2.37	3.97
			**19.20**	**7.43**	**15.90**
		61	1.43	999.28	184.77
			**1.28**	**1625.05**	**372.43**
		62	8.00	0.46	8.00
			**0.77**	**n.a.**	**63.90**
		63	0.00	0.98	0.70
			**n.a.**	**3.01**	**n.a.**
		64	0.00	0.00	2.10
			**n.a.**	**n.a.**	**28.82**
		65	1.72	1659.79	808.35
			**11.93**	**4690.98**	**3147.88**
		66	0.00	88.03	33.44
			**0.00**	**29.87**	**71.67**
		67	0.35	9.36	9.80
			**0.27**	**4.25**	**3.67**
		68	0.00	4.11	70.96
			**n.a.**	**7.59**	**57.75**
		69	0.00	189.23	16.42
			**n.a.**	**129.50**	**30.96**
		70	0.12	3.21	n.a.
			**0.18**	**8.38**	**n.a.**
		71	0.00	32.60	19.38
			**n.a.**	**77.64**	**39.74**
		72	0.00	1.13	49.54
			**0.00**	**6.72**	**227.29**
		73	0.88	33.35	184.18
			**4.94**	**139.98**	**683.95**
		74	0.56	13.08	11.73
			**4.68**	**24.74**	**9.50**
		75	264.28	85.94	183.85
			**1315.97**	**717.96**	**1969.04**
		76	0.34	5.89	12.22
			**1.52**	**50.43**	**56.03**
		77	2.96	44.31	50.67
			**10.87**	**278.25**	**282.91**
		78	n.a.	0.00	0.00
			**n.a.**	**n.a.**	**n.a.**
		79	19.38	616.82	74.82
			**n.a.**	**2609.35**	**222.93**
		80	0.00	0.26	0.44
			**n.a.**	**0.85**	**0.35**

**Figure 2:**
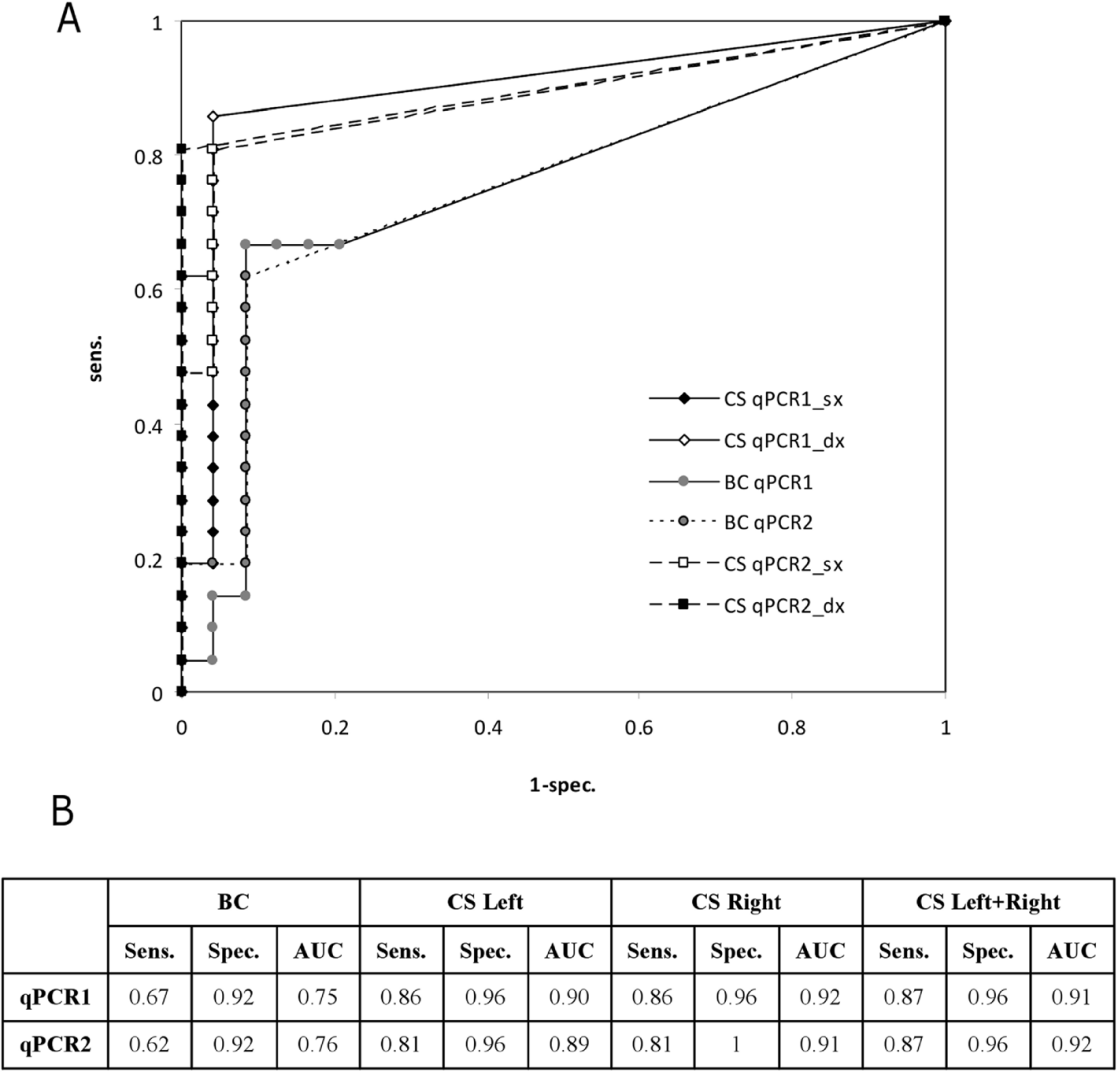
**Sensitivity and specificity analyses of qPCR assays. A)** ROC curves related to qPCR1 and qPCR2 performed in BC samples and CS samples. **B)** Sensitivity, specificity and area under the curve (AUC) related to qPCR1 and qPCR2.

Sensitivity and specificity results of all qPCR assays were compared and were significantly higher in CS qPCR than in BC qPCR, regardless of the primers that were used (Figure 2). No significant differences in terms of sensitivity and specificity results were observed between qPCR1 and qPCR2 in CS or BC samples (Z tests for difference of proportion, with Bonferroni correction). Since qPCR1 and qPCR2 results were comparable, only qPCR1 results were taken into consideration for subsequent statistical analyses.

Pearson correlations between the quantitative load of *Leishmania k*DNA in CS and BCS or IFAT titres were calculated in group D. The CS qPCR1 results correlated moderately with IFAT titres (R = 0.59), but the correlation with BCS was very weak (R = 0.37), according to previous literature data [27].

### Evaluation of clinical chemistry and haematological parameters as first line detection of *Leishmania* infection

The clinical parameters shown in Table 1 were re-evaluated considering the CS qPCR-positive and negative dogs (n = 27 and n = 45, respectively), to identify the parameters related to the presence of *Leishmania* DNA and to evaluate the usage of these parameters as a first line diagnostic approach in suspected cases with borderline IFAT titres 1:40–1:80 (i.e. group B), and previously infected dogs (i.e. group C).

Logistic regression with backward stepwise elimination showed that only globulins (O.R. = 6.00; C.I. = 1.51-23.87) and haemoglobin (O.R = 6.01; C.I. = 1.26-28.56) were significantly associated with group membership. Nagelkerke R square was 0.39, showing a modest but significant goodness of fit. Logistic regression specificity and sensitivity values were 0.704 and 0.739, respectively. If both globulins and haemoglobin parameters are above and below the normality range, respectively, the probability to belong to the infected group is 80%, whereas when both clinical parameters are in normality range, the probability to belong to the infected group is 20%.

### Use of CS qPCR for therapy monitoring

The efficiency of the qPCR assays used in this study were calculated to evaluate the possibility of making a direct comparison between the Ct values of *Leishmania* kDNA and the canine B2M gene. The qPCR efficiencies were calculated using serial dilutions of two CS lysates (from dogs 21, 38) spiked with known amounts of *L. infantum* MHOM/TN/80/IPT1 DNA. The ΔCt between B2M (qPCR3) and *Leishmania* kDNA (qPCR1 and qPCR2) were plotted against dilutions of CS lysates spiked with *L. infantum* DNA. The slope of the trend lines was < 0.1, indicating comparable PCR efficiencies [33] (Additional file 4: Figure S2). Hence, we concluded that a ΔCt value between *Leishmania* kDNA and canine B2M gene could be used to evaluate the trend of therapy or illness progression in the same animal.

Four infected dogs (57, 67, 68, 72) diagnosed with leishmaniasis, entered a therapeutic protocol and were monitored for parasite load in CS samples during therapy. Three dogs resulted qPCR negative in left and right CS samples after initiation of therapy; one dog (number 67) resulted qPCR negative in the left CS, and presented a parasite reduction in the right CS. Notably, in this sample the decrease in parasite load was verifiable by the increase in the ΔCt Leish-B2M value (Table 3).

**Table 3:** **CS qPCR1 application for therapy monitoring**

**Dog ID**	Day*	**Left CS qPCR (par/5×10** ^**4**^ **cells)**	**Right CS qPCR (par/5×10** ^**4**^ **cells)**	**Left ΔCt**	**Right ΔCt**	**Therapy protocol**
57	0	0.00	1.83	--	4.51	Glucantime doxycycline
	17	0.00	0.00	--	--	
67	0	9.36	9.80	−0.25	−0.28	Miltefosine
	30	0.00	1.87	--	2.46	
68	0	4.11	70.96	1.05	−3.15	Glucantime allopurinol
	56	0.00	0.00	--	--	
72	0	1.13	49.54	3.67	−1.77	Glucantime allopurinol
	49	0.00	0.00	--	--	

*day 0 corresponds to initiation of therapy.

## Discussion

The development of non-invasive sampling techniques is a worthy goal in human and veterinary medicine. In addition, in veterinary medicine, even more than in human medicine, there is a need for rapid sampling, limiting the use of patient sedation. Conjunctival swabs appear to be a very useful non-invasive sampling method although the technique may have drawbacks regarding sensitivity [34] and quantification due to variability in the amount of biological material collected. To get around the variability issue the CS qPCR results can be normalized to the number of canine cells by parallel quantification of a single copy canine gene (e.g. β-actin) [26]. Several studies have focused on the usefulness of CS PCR in the initial diagnosis of CanL but little research has been done on its applicability in particular clinical cases.

The present study assessed the diagnostic value of CS qPCR in dogs with borderline IFAT titres, suspected disease relapse or the presence of comorbidity, comparing the CS qPCR results with BC qPCR and haematochemical analyses. The use of BC samples to detect *Leishmania* in dogs is a much debated issue [35-37]. Our study confirmed that BC qPCR is not as accurate as CS qPCR as a tool to rule out suspected leishmaniasis infection. However, BC qPCR could be useful for CanL diagnosis confirmation, and/or for blood donor dog screening, as we observed in the cases of dogs 4 and 12 in this investigation. At the time of screening, these dogs were IFAT negative and without clinical symptoms, but both resulted BC qPCR positive (with a middle-high load). Only dog 4 was also CS qPCR positive. Ten months after the biomolecular positivity, the IFAT was borderline with a titre of 1:40 but still no symptomatology. This highlights the importance of biomolecular analysis in blood donor screening because these dogs were suspended from donor program only after testing positive with the BC qPCR. In fact, *Leishmania* DNA positivity without symptomatology may reflect seasonal contact with the infected vector and does not necessarily involve the appearance of specific symptoms also in the presence of borderline IFAT titres [10,38]. BC qPCR positivity with no clinical signs also occurs in humans. Specifically, the transition from biomolecular positive to negative results has been observed in pediatric patients without symptomatology [35].

In order to obtain quantitative data from CS samples, the parasite load was normalized using the B2M gene and the results were expressed per 50,000 canine cells. The normalization can overcome the problem due to the intrinsic variability of the swab sampling and would be helpful to compare data from different laboratories also because of the non-standardized way of collecting conjunctive cells using swabs. This procedure allowed us to quantify the CS parasite load and to monitor the same animal during therapy; however, it increased the cost of analysis and required more time. This disadvantage was partially reduced by using SYBR Green and CS raw lysate as the source of the DNA template. In fact, the use of CS raw lysate, instead of DNA extracted with the phenol-chloroform method [23,26,39] or with commercial kits [25,27], enabled us to obtain good sensitivity (1×10^−3^ par/reaction tube), at a low cost and without inhibitory effects when 1 μl per reaction tube was used. We found a CS-qPCR sensitivity and specificity of 87% and 96%, respectively, confirming and improving on the results obtained by Gramiccia *et al.* [29]. These data were also in agreement with other previous investigations [23,26] although those authors used the phenol-chloroform method.

Interestingly, among the clinical and haematological parameters considered, those most associated with qPCR positive results were globulins and haemoglobin. This finding seems to suggest that these parameters in particular should be considered during first-line laboratory tests in the case of suspected CanL or evaluation of disease relapse. However, even considering these parameters, relevant uncertainty remained, supporting the need of a wider use of qPCR.

The present study also investigated the use of CS qPCR in borderline and disease relapse cases with complex diagnosis, including comorbidity. In group B (borderline cases) only 2 dogs (27 and 40) out of 17 tested positive with CS qPCR. Dog 27 showed a very low parasite load in the left CS (0.05 par/50,000 cells). The BCS was 1, IFAT titre was 1:40 and serum electrophoresis and biochemical analysis were normal; however the haemochrome showed a moderate anaemia compatible with leishmaniasis. Prina *et al.* [40] demonstrated that *Leishmania* DNA is rapidly degraded following parasite death; hence, its identification in conjunctival tissue could be indicative of recent extraepidermical dissemination that, in presence of clinical signs or, as in this case, haematological signs, could require treatment or close monitoring. Another complicated case study, in terms of therapeutic decisions, was dog 40 because of the presence of comorbidity. This dog was in therapy for hypothyroidism when *Leishmania* was detected. Subsequently, *Dirofilaria* spp., *Trichuris* spp. and *Toxocara* spp. were also diagnosed by optical microscopy. There were no significant alterations in biochemical and haematological parameters but the BCS was 8. Regarding the qPCR positive results, although indicating low parasite load, the veterinary practitioner started the animal on a canine *Leishmania* therapy with allopurinol alone and specific therapy for the other parasitosis mentioned above. An evident improvement in symptoms was reported four months later.

Furthermore, eight dogs (42, 43, 44, 45, 46, 48, 50, 57) out of 16 in group C (monitored after therapy) tested positive in at least one of the BC and CS samples analyzed. It is noteworthy that only dog 42 had important alterations in its biochemical values; 6 out of 6 with available data had alterations in their electrophoretic patterns (Additional file 2: Table S2) and 4 out of 7 with available data had anemia or thrombocytopenia (the hemogram of dog 50 was not available). These data suggest that qPCR is a very important and specific tool in the monitoring of disease relapse. In fact, various comorbidities were found in dogs 42, 43, 45 and 46. These comorbidities potentially interfered with the electrophoresis panel, but the positive qPCR confirmed the suspicion of a leishmaniasis relapse.

Many authors have suggested the use of biomolecular approaches in therapy monitoring, observing that a residual parasite load is normally found in samples from lymph-nodes or bone marrow after therapeutic protocols [15]. This could complicate the assessment of therapeutic response in some cases. Hence, we also investigated the potential role of CS in therapy monitoring. A parasite load = 0 in dogs under the treatment (group E) was found in 3 out of 4 dogs tested during their therapy, while the dog 67 presented a parasite reduction in the right CS, which was reflected by the increase in the ΔCt Leish-B2M value. Despite the low number of animals, this could furnish a proof of concept that this relative approach could be used, avoiding parasite load absolute quantification.

## Conclusions

In conclusion, this study has shown the practical application of a qPCR assay in canine leishmaniasis diagnosis, which can be used together with conventional assays, providing valuable additional information for veterinary therapeutic choices. Moreover, our investigation also suggests that CS qPCR is not only useful for first initial diagnosis but also provides useful information for therapeutic decisions in borderline cases and in disease relapse re-evaluation. Furthermore, the data showed that, in the conventional laboratory analyses that were considered, the values of globulins and haemoglobin were more strongly associated with CS qPCR positivity.

Lastly, the equivalence of qPCR1 and qPCR2 was verified, and a different method of therapy monitoring, independent of parasite load quantification, was introduced. In fact, calculating the ΔCt Leish-B2M, it was possible to obtain a value that should increase during an effective therapy or diminish/remain stable in the case of therapy failure or disease relapse. This approach could get around the problem of exact parasite load quantification (based on standard reference strains) and relative normalization.

On the whole, the CS qPCR results obtained in dogs suggest more extensive investigation of the use of this kind of samples in human cases also.

## Additional files

Additional file 1: Table S1. Clinical parameters for all animals. Additional file 2: Table S2. Serum electrophoresis results. Additional file 3: Figure S1. B2M calibration curve. The curve was obtained by 10-fold serial dilution of purified PCR product ranging from 19.4 to 19.4 × 10^7^ molecules of B2M PCR product. Additional file 4: Figure S2. Relative efficiency plot of B2M (qPCR3) and *Leishmania* kDNA amplified with qPCR1 (A) and qPCR2 (B). ΔCt values (Ct *Leishmania* kDNA - Ct B2M) were plotted against log of dilutions of CS lysate samples spiked with *L. infantum* DNA (equivalent to 1 × 10^3^ par/μl).

## Abbreviations

CS: Conjunctival swab
BC: Buffy coat
qPCR: Quantitative real-time PCR
VL: Visceral leishmaniasis
CL: Cutaneous leishmaniasis
CanL: Canine leishmaniasis
IFAT: Indirect Fluorescence Antibody Test
BCS: Body condition score
B2M: Beta-2-microglobulin
kDNA: Kinetoplast DNA
ROC: Receiver operating characteristic
