# Supplementary material for: Application of qPCR in conjunctival swab samples for the evaluation of canine leishmaniasis in borderline cases or disease relapse and correlation with clinical parameters
Source: Parasit Vectors. 2014 Oct 21;7:460. doi: 10.1186/s13071-014-0460-3 (PMC4207623; doi:10.1186/s13071-014-0460-3)
Supplement: Additional file 1: Table S1. — Clinical parameters for all animals. [file 13071_2014_460_MOESM1_ESM.pdf]

Table S1. **Clinical parameters for all animals**. BCS: body condition score; Prot: total proteins; Alb: albumins; Glob: globulins; WBC: white blood cells; RBC: red blood cells; Hgb: haemoglobin; HCT: haematocrit; Plt: platelets

Group	Dog ID	IFAT	BCS	Prot (g/dl)	Alb (g/dl)	Glob (g/dl)	ratio Alb/Glob	WBC $10^3/\mu l$	RBC $10^6/\mu l$	Hgb (g/dl)	Hct (%)	Plt $10^3/\mu l$
A	1	<1/40	0	6.46	3.90	2.56	1.52	12.6	6.44	16.5	45.3	255
	2	<1/40	1	n.a.	n.a.	n.a.	n.a.	62.0	2.40	6.3	19.2	201
	3	<1/40	0	6.26	3.70	2.56	1.45	10.9	5.76	15.5	42.0	227
	4	<1/40	0	6.21	3.60	2.61	1.38	11.3	6.17	16.3	43.6	347
	5	<1/40	0	6.59	3.20	3.39	0.94	10.9	5.81	14.7	39.1	204
	6	<1/40	0	n.a.	n.a.	n.a.	n.a.	7.4	5.18	14.1	38.6	240
	7	<1/40	0	6.21	3.60	2.61	1.38	11.8	5.96	16.1	43.5	307
	8	<1/40	0	6.17	3.40	2.77	1.23	17.3	5.25	14.2	38.2	394
	9	<1/40	0	5.67	3.10	2.57	1.21	11.7	5.35	15.6	38.9	198
	10	<1/40	0	5.70	2.64	3.06	0.86	n.a.	n.a.	n.a.	n.a.	n.a.
	11	<1/40	0	6.70	3.22	3.48	0.92	n.a.	n.a.	n.a.	n.a.	n.a.
	12	<1/40	0	6.78	3.60	3.18	1.13	11.0	6.13	15.4	44.7	235
	13	<1/40	0	n.a.	n.a.	n.a.	n.a.	n.a.	n.a.	n.a.	n.a.	n.a.
	14	<1/40	1	n.a.	n.a.	n.a.	n.a.	14.6	7.32	18.1	51.6	243
	15	<1/40	3	n.a.	n.a.	n.a.	n.a.	n.a.	n.a.	n.a.	n.a.	n.a.
	16	<1/40	2	6.86	4.00	2.86	1.40	9.3	4.94	11.4	31.2	468
	17	<1/40	3	6.73	2.80	3.93	0.71	10.1	5.74	13.8	37.8	361
	18	<1/40	0	n.a.	n.a.	n.a.	n.a.	n.a.	n.a.	n.a.	n.a.	n.a.
	19	<1/40	3	6.91	3.40	3.51	0.97	n.a.	n.a.	n.a.	n.a.	n.a.
	20	<1/40	1	6.19	3.30	2.89	1.14	13.2	7.87	20.3	52.9	273
	21	<1/40	0	7.96	3.50	4.46	0.78	12.7	5.74	14.6	37.5	470
	22	<1/40	1	5.79	3.70	2.09	1.77	14.2	6.78	19.5	48.6	255
	23	<1/40	8	7.06	3.7	3.36	1.1	26.5	5.66	16.1	41.3	280
	24	<1/40	2	n.a.	n.a.	n.a.	n.a.	8.6	6.17	16.6	46.6	313
B	25	1:80	0	n.a.	n.a.	n.a.	n.a.	13.5	5.09	12.1	37.1	284
	26	1:40	1	8.16	2.90	5.26	0.55	23.0	6.47	15.0	45.0	372
	27	1:40	0	6.10	3.12	2.98	1.06	11.8	4.3	11.1	31.1	324
	28	1:40	2	7.78	3.00	4.78	0.63	8.3	5.36	14.9	35.8	432
	29	1:40	0	n.a.	n.a.	n.a.	n.a.	n.a.	n.a.	n.a.	n.a.	n.a.
	30	1:80	1	6.50	2.84	3.66	0.78	n.a.	n.a.	n.a.	n.a.	n.a.
	31	1:80	1	6.58	3.00	3.58	0.83	9.3	5.02	13.7	37.1	348
	32	1:40	3	6.86	3.10	3.76	0.82	9.2	5.10	13.8	38.3	367
	33	1:40	0	n.a.	n.a.	n.a.	n.a.	n.a.	n.a.	n.a.	n.a.	n.a.
	34	1:80	0	7.96	3.50	4.46	0.78	12.7	5.74	14.6	37.5	470
	35	1:40	1	n.a.	n.a.	n.a.	n.a.	n.a.	n.a.	n.a.	n.a.	n.a.
	36	1:40	1	5.86	2.90	2.96	0.98	6.2	5.39	13.4	39.4	185
	37	1:80	2	n.a.	n.a.	n.a.	n.a.	n.a.	n.a.	n.a.	n.a.	n.a.
	38	1:40	2	6.34	2.80	3.54	0.79	9.2	5.74	15.1	40.7	456

	39	1:80	4	n.a.	n.a.	n.a.	n.a.	n.a.	n.a.	n.a.	n.a.	n.a.
	40	1:80	8	6.49	3.00	3.49	0.86	16.3	6.90	18.3	45.6	243
	41	1:40	5	n.a.	n.a.	n.a.	n.a.	12.0	5.2	14.4	33.9	342
C	42	n.a.	0	8.35	3.20	5.15	0.62	7.0	6.08	14.9	43.7	346
	43	n.a.	1	6.16	1.90	4.26	0.45	8.8	3.68	9.2	27.9	343
	44	1:160	4	7.09	2.80	4.29	0.65	28.7	3.49	9.4	24.2	298
	45	n.a.	2	6.86	3.00	3.86	0.78	9.9	6.14	16.9	43.4	106
	46	n.a.	2	6.11	2.80	3.31	0.85	4.2	5.57	14.0	38.7	223
	47	1:320	1	6.67	3.10	3.57	0.87	8.5	7.14	19.4	47.3	523
	48	n.a.	1	7.00	3.60	3.40	0.88	n.a.	n.a.	n.a.	n.a.	n.a.
	49	1:320	0	6.30	2.95	3.35	0.89	n.a.	n.a.	n.a.	n.a.	n.a.
	50	1:320	1	6.00	2.34	3.66	0.64	n.a.	n.a.	n.a.	n.a.	n.a.
	51	1:160	0	5.83	3.30	2.53	1.30	12.9	6.04	17.0	43.3	231
	52	1:320	2	6.35	3.10	3.25	0.95	8.3	6.14	17.0	43.7	173
	53	1:1280	4	n.a.	n.a.	n.a.	n.a.	4.7	6.77	17.8	50.7	238
	54	1:160	2	6.26	3.10	3.16	0.98	4.6	5.43	15.1	39.3	284
	55	1:80	0	n.a.	n.a.	n.a.	n.a.	n.a.	n.a.	n.a.	n.a.	n.a.
	56	1:160	1	6.03	2.90	3.13	0.93	9.8	6.06	16.9	43.1	220
	57	1:640	6	7.80	3.90	3.90	1.00	11.1	0.92	2.7	7.4	112
D	58	1:320	3	7.13	2.20	4.93	0.45	10.1	5.77	14.0	39.8	309
	59	1:320	0	7.83	2.60	5.23	0.50	10.0	5.47	13.7	38.0	229
	60	1:640	1	n.a.	n.a.	n.a.	n.a.	n.a.	n.a.	n.a.	n.a.	n.a.
	61	1:5120	1	10.84	2.30	8.52	0.27	6.2	4.33	9.2	24.0	172
	62	1:640	3	7.99	2.80	5.19	0.54	12.9	6.76	16.7	45.7	200
	63	1:2560	2	6.34	2.60	3.74	0.70	18.2	6.13	14.6	43.2	198
	64	1:640	0	6.50	3.09	3.41	0.91	n.a.	n.a.	n.a.	n.a.	n.a.
	65	1:5120	2	8.50	0.96	7.54	0.13	13.7	4.14	9.4	27.2	127
	66	1:2560	5	n.a.	n.a.	n.a.	n.a.	10.6	6.53	15.2	44.3	307
	67	1:5120	1	6.97	2.00	4.97	0.40	3.9	1.94	5.5	15.0	94
	68	1:640	2	8.14	2.70	5.44	0.50	7.5	4.88	11.8	32.2	383
	69	1:320	6	8.79	1.90	6.89	0.50	3.2	3.08	8.5	22.2	67
	70	1:2560	3	10.28	2.70	7.58	0.36	9.7	4.85	11.9	32.7	127
	71	1:640	3	9.01	3.30	5.71	0.58	7.0	6.00	14.8	43.7	196
	72	1:2560	4	7.52	2.40	5.12	0.47	8.2	3.30	8.0	25.1	124
	73	1:10240	8	9.38	1.70	7.68	0.22	12.9	5.71	12.4	38.8	214
	74	1:2560	11	7.96	2.00	5.96	0.34	19.0	3.64	9.5	25.6	275
	75	1:1280	6	9.97	2.70	7.27	0.37	12.1	4.63	12.0	31.1	188
	76	1:160	6	n.a.	n.a.	n.a.	n.a.	n.a.	n.a.	n.a.	n.a.	n.a.
	77	1:2560	5	9.9	2.9	7	0.41	7.8	6.07	11.8	35.2	324
	78	1:160	7	5.6	3.7	1.90	1.95	16.5	6.87	17.9	48.7	364
	79	1:1280	3	6.84	2.5	4.34	0.58	17.4	5.19	12.8	39.5	47
	80	1:160	1	n.a.	n.a.	n.a.	n.a.	n.a.	n.a.	n.a.	n.a.	n.a.

n.a.: not available
