# Supplementary material for: Application of qPCR in conjunctival swab samples for the evaluation of canine leishmaniasis in borderline cases or disease relapse and correlation with clinical parameters
Source: Parasit Vectors. 2014 Oct 21;7:460. doi: 10.1186/s13071-014-0460-3 (PMC4207623; doi:10.1186/s13071-014-0460-3)
Supplement: Additional file 2: Table S2. — Serum electrophoresis results. [file 13071_2014_460_MOESM2_ESM.pdf]

Table S2. **Serum electrophoresis results**. Data from 44 dogs are reported.

Group	Dog ID	Alb fraction %	α 1 fraction %	α 2 fraction %	β fraction %	γ fraction %
A	1	49.4	3.2	16	22.8	8.6
	2	46.7	5.6	15.4	25.5	6.8
	3	46.6	3	14.6	24.7	11.1
	4	49.9	4.1	15.1	23.2	7.7
	5	52.8	2.9	14.1	20.8	9.4
	7	47.3	3.6	16	24.4	8.7
	8	50.5	2.6	18.1	21.5	7.3
	10	46.2	4.1	21.2	21.3	7.2
	11	48	2.6	18.2	24.5	6.7
	12	43.1	3.3	14.1	29.1	10.4
	16	46.2	3	18.1	24.3	8.4
	24	55.6	3.4	13.5	17.6	9.9
B	26	34.3	1.4	14.6	35.4	14.3
	27	51.4	3.2	19.5	19.5	6.4
	28	30.8	1.9	19.2	24.5	23.6
	30	43.9	3.8	18.7	26.9	6.7
	31	45.4	2.3	13.4	26.3	12.8
	32	52.6	2.4	7.9	26.3	10.8
	36	51.5	2.4	12.9	21.6	11.6
	39	40.6	1.8	7.6	36.3	13.7
C	42	11.8	1.5	9.1	12.3	65.3
	43	16.3	1.1	15.6	30.9	36.1
	44	33.5	3.6	9.8	43.5	9.6
	45	33.7	2.1	29.9	27.4	6.9
	46	32	3.2	12.4	37.4	15
	47	36.1	1.7	29.6	23.2	9.4
	49	47	3.1	16.3	25.4	8.2
	50	38.9	2.9	21.9	28	8.3
	51	50.8	3.9	12.8	22.8	9.7
	52	46.9	2.4	12.7	27.6	10.4
D	58	10.6	1.2	9.5	20.6	58.1
	59	29.8	3.4	7.9	14	44.9
	61	23.9	1.6	14.7	30.8	29
	62	9.4	1.4	7.4	14.4	67.4
	63	39.5	1.2	24.4	21	13.9
	64	47.6	3.2	13	24.4	11
	65	11.3	0.8	7.6	23.9	56.4
	67	23	2.1	15.2	28.3	31.4
	69	12.1	1.3	3.2	16.8	66.6
	71	28.4	1.2	9.6	18.2	42.6
	72	19.5	1.2	6.9	9.4	63
	73	9.3	0.6	4.6	12.6	72.9
	74	12.8	0.9	9	17.3	60
	75	8.6	1.7	12.9	36	40.8
