# Supplementary figures and images for: Application of qPCR in conjunctival swab samples for the evaluation of canine leishmaniasis in borderline cases or disease relapse and correlation with clinical parameters
Source: Parasit Vectors. 2014 Oct 21;7:460. doi: 10.1186/s13071-014-0460-3 (PMC4207623; doi:10.1186/s13071-014-0460-3)

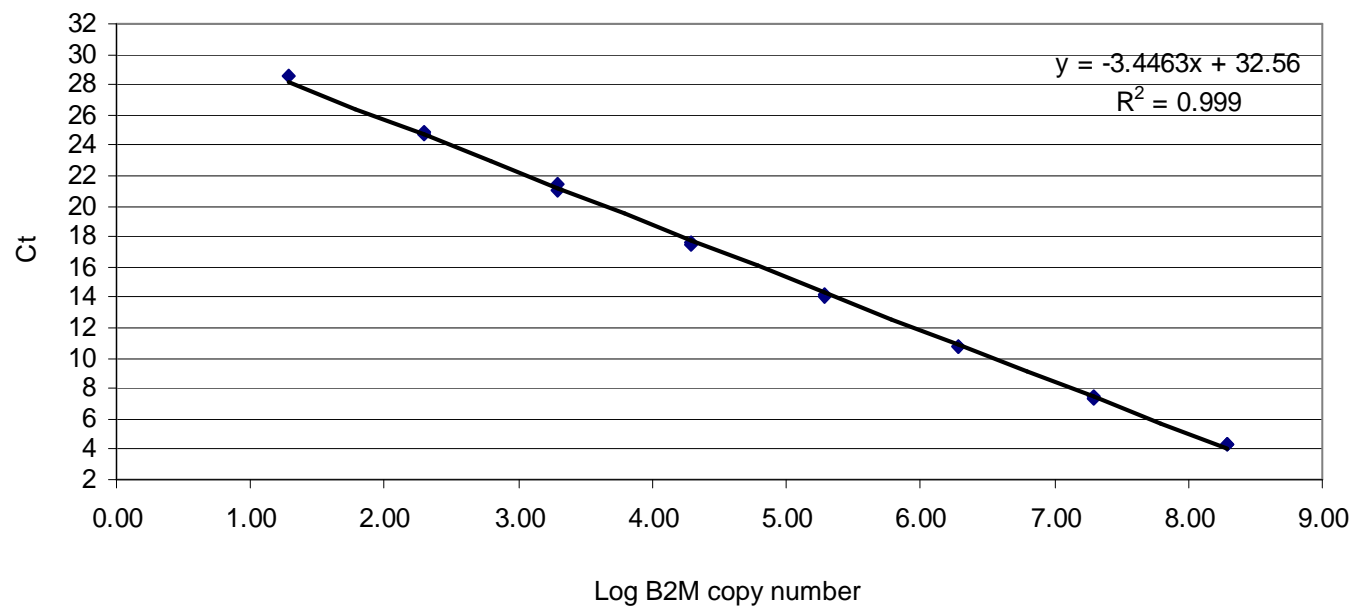

Supplement: Additional file 3: Figure S1. — B2M calibration curve. The curve was obtained by 10-fold serial dilution of purified PCR product ranging from 19.4 to 19.4 × 107 molecules of B2M PCR product. [file 13071_2014_460_MOESM3_ESM.pdf]

A

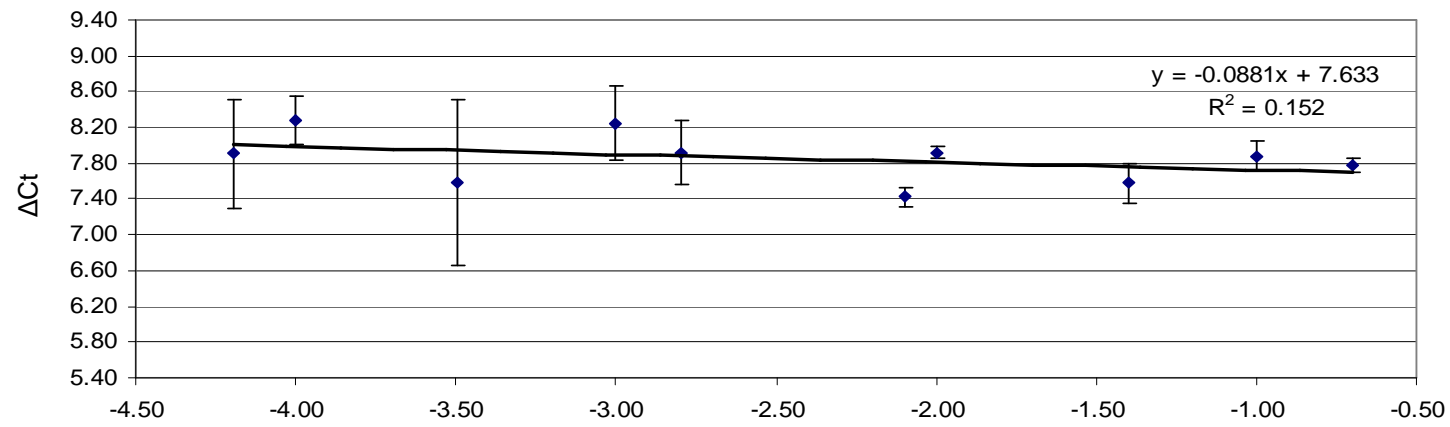

B

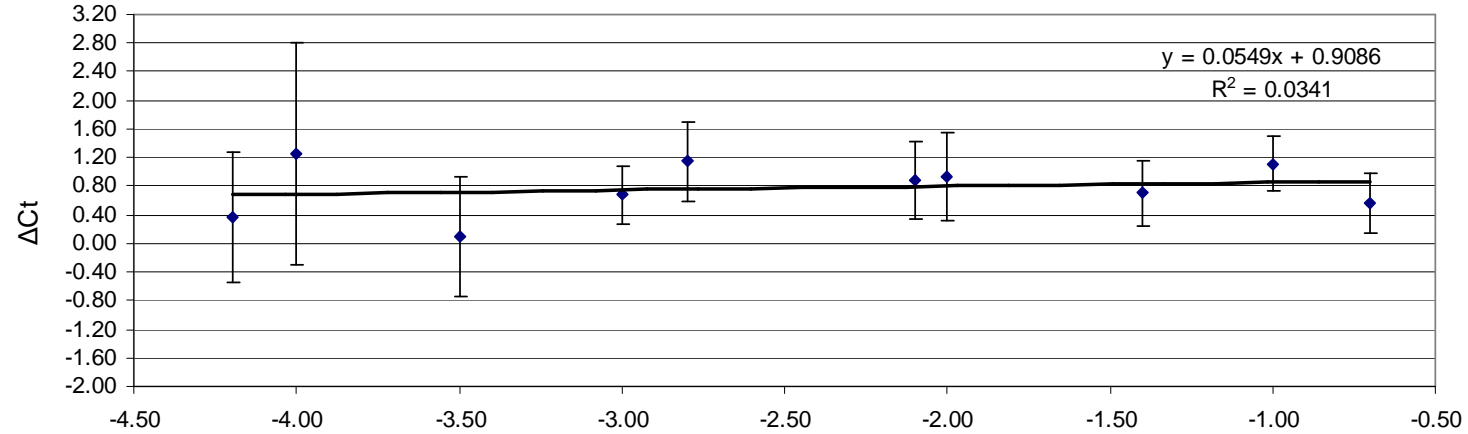

Log dilutions of CS lysates spiked with *L. infantum* DNA

Supplement: Additional file 4: Figure S2. — Relative efficiency plot of B2M (qPCR3) and Leishmania kDNA amplified with qPCR1 (A) and qPCR2 (B). ΔCt values (Ct Leishmania kDNA - Ct B2M) were plotted against log of dilutions of CS lysate samples spiked with L. infantum DNA (equivalent to 1 × 10^3^ par/μl). [file 13071_2014_460_MOESM4_ESM.pdf]
